# Phenotypic Mutation Rates and the Abundance of Abnormal Proteins in Yeast

**DOI:** 10.1371/journal.pcbi.0030203

**Published:** 2007-11-23

**Authors:** Martin Willensdorfer, Reinhard Bürger, Martin A Nowak

**Affiliations:** 1 Program for Evolutionary Dynamics, Harvard University, Cambridge, Massachusetts, United States of America; 2 Department of Molecular and Cellular Biology, Harvard University, Cambridge, Massachusetts, United States of America; 3 Department of Mathematics, University of Vienna, Vienna, Austria; 4 Department of Mathematics, Department of Organismic and Evolutionary Biology, Harvard University, Cambridge, Massachusetts, United States of America; University of Texas Austin, United States of America

## Abstract

Phenotypic mutations are errors that occur during protein synthesis. These errors lead to amino acid substitutions that give rise to abnormal proteins. Experiments suggest that such errors are quite common. We present a model to study the effect of phenotypic mutation rates on the amount of abnormal proteins in a cell. In our model, genes are regulated to synthesize a certain number of functional proteins. During this process, depending on the phenotypic mutation rate, abnormal proteins are generated. We use data on protein length and abundance in Saccharomyces cerevisiae to parametrize our model. We calculate that for small phenotypic mutation rates most abnormal proteins originate from highly expressed genes that are on average nearly twice as large as the average yeast protein. For phenotypic mutation rates much above 5 × 10^−4^, the error-free synthesis of large proteins is nearly impossible and lowly expressed, very large proteins contribute more and more to the amount of abnormal proteins in a cell. This fact leads to a steep increase of the amount of abnormal proteins for phenotypic mutation rates above 5 × 10^−4^. Simulations show that this property leads to an upper limit for the phenotypic mutation rate of approximately 2 × 10^−3^ even if the costs for abnormal proteins are extremely low. We also consider the adaptation of individual proteins. Individual genes/proteins can decrease their phenotypic mutation rate by using preferred codons or by increasing their robustness against amino acid substitutions. We discuss the similarities and differences between the two mechanisms and show that they can only slow down but not prevent the rapid increase of the amount of abnormal proteins. Our work allows us to estimate the phenotypic mutation rate based on data on the fraction of abnormal proteins. For S. cerevisiae, we predict that the value for the phenotypic mutation rate is between 2 × 10^−4^ and 6 × 10^−4^.

## Author Summary

A functional protein machinery, built from genetic information, is central to every living organism. Surprisingly, the decoding of genes into amino acid sequences is fairly inaccurate. Errors in this process (phenotypic mutations) are several orders of magnitude more frequent than errors during DNA replication (genotypic mutations). Many researchers have explored the evolution of genotypic mutation rates, but there are as yet few investigations into the evolutionary dynamics of phenotypic mutation rates. Here we present a mathematical model that describes the effect of phenotypic mutation on the amount of abnormal proteins in cells. We parameterize our model using data from yeast (Saccharomyces cerevisiae). We show that for phenotypic mutation rates above 5 × 10^−4^ per amino acid, the error-free synthesis of large proteins becomes nearly impossible. We estimate the phenotypic mutation rate of S. cerevisiae to be between 2 × 10^−4^ and 6 × 10^−4^ per amino acid.

## Introduction

Every biological organism is built according to information stored in its genome. Genomes composed of billions of base pairs are not unusual. This information has to be duplicated during cell replication. Since replication errors can have devastating effects, DNA replication needs to be very accurate. Estimates for error rates in Eukaryotes are as low as 5 × 10^−4^ errors per base pair per replication [1]. But even flawless genetic information is useless if the cell is not able to synthesize functional proteins. Transcription and translation, the two processes involved in decoding DNA, have to be sufficiently accurate to allow a cell to build a reliable protein machinery. We refer to errors that occur during transcription and translation as phenotypic errors, and to errors that occur during DNA replication as genotypic errors. Most phenotypic errors are introduced during translation when ribosomes translate RNA sequences into amino acid sequences [2,3]. The accuracy of translation depends on the considered codon and context. In Escherichia coli it can range from 5 × 10^−4^ to 1 × 10^−4^ (see Table 1 for some examples), with 5 × 10^−4^ as a commonly used estimate for the average frequency of errors per codon [4,5]. In comparison, Blank et al. [2] measured an E. coli error rate during transcription of 5 × 10^−6^.

**Table 1:**
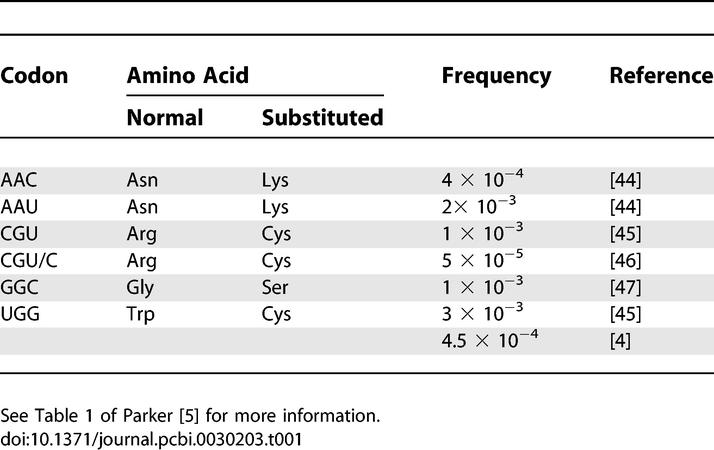
Amino Acid Substitution Rates During Protein Synthesis in E. coli

Measuring the genotypic mutation rate is easier than measuring the phenotypic mutation rate. Estimates of genotypic mutation rates exist for many organisms. The data show that the number of mutations per genome per replication is constant for a wide range of organisms [1]. This is in agreement with theoretical results that suggest that the number of errors per replication per genome have to be below a certain error threshold to avoid an error catastrophe at which the propagation of genetic information becomes impossible [6–9]. There are many theoretical approaches for studying the evolution of genotypic mutation rates [10–14], and one can tentatively claim that we have a basic understanding of what governs the evolution of genotypic mutation rates.

This is not the case for phenotypic mutation rates. Apparently, very little theoretical work has been devoted to this topic. A notable exception is Wilke and Drummond [15], who study translational robustness and the evolution of gene-specific phenotypic mutation rates. Their work predicts selection for proteins that fold properly despite mistranslation and provides an explanation for the fact that highly expressed genes evolve slower. A closely related study, and the starting point for this investigation, is Bürger et al. [16]. They studied a model in which the total number of synthesis attempts to produce sufficiently many functional proteins is limited and showed that the selection pressure to reduce the phenotypic mutation rate below a certain threshold vanishes.

In addition, empirical information is scarce. Most measurements of phenotypic mutation rates are limited to E. coli [5]. For fast-growing E. coli laboratory strains, a correlation was found between ribosomal accuracy and ribosomal kinetics [3,17]. This suggests that the (high) phenotypic mutation rates are a result of a cost–benefit tradeoff. More accurate ribosomes reduce speed of translation and are hence disadvantageous. Natural isolates, however, do not show such a correlation between ribosomal accuracy and ribosomal kinetics. They display a wide diversity of ribosomal kinetic properties and growth rates which suggests that the tradeoff between accuracy and kinetics is not limiting in natural populations [17–19]. Apparently, natural populations are not so obsessed with optimizing translation kinetics for fast growth under laboratory conditions. This is not surprising considering that the estimated doubling time of, for example, intestinal E. coli (40 h) is substantially longer than the doubling time of laboratory strains (0.5 h) [20].

Hence, it is not clear if the optimization of translation kinetics is governing the evolution of phenotypic mutation rates. In this paper we analyse phenotypic mutations from a genomic/proteomic viewpoint. In particular, we derive and analyze a model that allows us to calculate the amount of abnormal proteins in a cell as a function of the phenotypic mutation rate. We also evolve genotypic and phenotypic mutation rates in computer simulations that are based on properties of the Saccharomyces cerevisiae genome/proteome. We discover that the current estimate for global phenotypic mutation rates of 5 × 10^−4^ is at a value where the amount of erroneous proteins begins to increase exponentially with the mutation rate. Further, at this value we observe a change in the kind of genes that contribute the most to erroneous proteins. For phenotypic mutation rates below 5 × 10^−4^, erroneous proteins from highly expressed genes are frequent. Above 5 × 10^−4^, however, erroneous proteins from large genes begin to dominate. Finally, we study models in which individual proteins can decrease their phenotypic mutation rate by using preferred codons or evolve robustness against amino acid substitutions. We point out the similarities and differences between the two mechanisms and show how an increase of the amino acid substitution rate above 5 × 10^−4^ affects the adaptation of highly expressed proteins.

## Materials and Methods

In the following, we develop and analyze a model regarding the evolution of phenotypic mutation rates. We use data from S. cerevisiae to parameterize our model and calculate here relevant properties of the available yeast data.

The genotypic mutation rate in S. cerevisiae is approximately 2.2 × 10^−10^ mutations per base pair per replication [1]. Our model requires the number of deleterious mutations per codon per replication as the unit for the genotypic mutation rate. Since each codon is composed of three nucleotide acids and 438/576 ≈ 3/4 single site mutations are nonsynonymous [21], the mutation rate per codon is given by 3 × 3/4 × 2.2 × 10^−10^ = 4.95 × 10^−10^. Of these nonsynonymous mutations, about 10% to 60% [22] are deleterious, placing the genotypic mutation rate somewhere between 4.95 × 10^−11^, and 2.97 × 10^−10^ deleterious mutations per codon per replication. For simplicity, we use 1 × 10^−10^. The phenotypic mutation rate in yeast appears to be similar to the mutation rates measured in E. coli [23]. The mutation rate is therefore likely to range from 1 × 10^−5^ to 5 × 10^−3^, with 5 × 10^−4^ as an estimate for the global phenotypic mutation rate [5].

To parameterize our model, we need the length *n_i_* and abundance *y_i_* of each protein in yeast. Complete genomic sequences [24] provide the length, *n_i_*, of each protein of an organism. We only consider reading frames from the *Saccharomyces* Genome Database [24] that have been classified as nonspurious by Ghaemmaghami et al. [25]. This leaves us with 5,675 open reading frames (proteins) that have an average length of 496 amino acids. The effective genome is 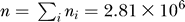 residues long. Information about the abundance, *y_i_*, of proteins is provided by Ghaemmaghami et al. [25]. From their data we calculate that the total amount (measured in number of amino acids) of functional proteins is given by 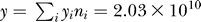 and that 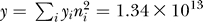 (this expression is relevant for Equation 8).

## Results

### The Model

We consider a large population of asexual single-cell organisms (cells, for short), each with one DNA chromosome and *K* genes of (possibly) different length and expression level. Gene *i* (*i* = 1,…,*K*) is *n_i_* amino acids long. During one cell cycle, *y_i_*, functional proteins have to be synthesized from gene *i*. We assume that regulation of gene expression guarantees that the gene is expressed until *y_i_* functional proteins are present. A cell with error-free transcription and translation will therefore synthesize exactly *y_i_* proteins of gene *i*. Since we are interested in variation within a population, we can normalize the fitness of such a cell to 1 and consider only relative fitnesses. Any cost of synthesizing all the functional proteins is accounted for in the fitness value of 1. Cells, however, have a phenotypic mutation rate *u* > 0, which denotes the probability (per codon) that protein synthesis is erroneous and produces a nonfunctional protein. Hence, a phenotypic mutation is a deleterious amino acid substitution that occurs during protein synthesis. We assume that phenotypic mutations are independent of each other. Hence, the probability of synthesizing a nonfunctional protein from gene *i* is given by 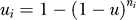 . Let *x_i_* denote the number of nonfunctional proteins that have been synthesized until *y_i_* functional proteins were made. Then a cell synthesizes 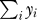 functional and 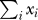 nonfunctional proteins. It uses 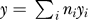 amino acids to synthesize functional proteins and 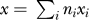 amino acids to synthesize nonfunctional proteins.

We are interested in the cost of erroneous protein synthesis in natural populations. Natural populations grow much slower than laboratory cultures. The growth of bacteria is limited by the rate of protein synthesis per ribosome. In slow-growing bacteria, the rate of protein synthesis per ribosome is, because of limiting amounts of charged tRNAs, almost 50% lower than in fast-growing bacteria [26]. We will therefore base our cost function on the effects of the phenotypic mutation rate on the availability of charged tRNAs.

Most erroneous proteins are identified as abnormal and are degraded rapidly [27]. In this case, the amino acids that have been used to synthesize the erroneous proteins can be recycled to charge new tRNAs. This constant turnover, however, will diminish the pool of charged tRNAs by an amount depending on *x*. Hence, the cost of phenotypic mutations is a function of *x*. We will use *η*(*x*) to denote the cost of erroneous protein synthesis. Even though we have motivated *η*(*x*) by the cost of depleting the tRNA pool, it can account for other possible costs of erroneous proteins synthesis as well. Examples include toxic effects of aggregates of misfolded proteins, the waste of metabolic energy (ATP/GTP), or usage of the ribosomal machinery to synthesize nonfunctional instead of functional proteins. Overall, the fitness of a cell that uses *x* amino acids to synthesize nonfunctional proteins is given by 1 − *η*(*x*). It is unnecessary to explicitly consider the cost of protein synthesis of functional proteins, *c_y_*, because *y* is constant and, hence, 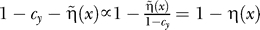 , where 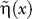 denotes the costs if the costs of synthesis of functional proteins are not included.

Besides phenotypic, we also take genotypic mutations into account. This allows us to directly compare the cost and evolution of phenotypic and genotypic mutation rates. Genotypic mutations introduce deleterious mutations at rate *μ* per codon, that is, gene *i* replicates successfully with probability 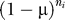 , and all genes are replicated successfully with probability (1 − *μ*)*^n^*, where 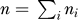 can be interpreted as the effective genome size, i.e., the total number of amino acids encoded by all *K* genes. We assume that the population is large enough so that deleterious mutations cannot spread. We ignore mutations that recover the wild type.

In the following, the average fitness of a population with phenotypic and genotypic mutation rates *u* and *μ* per codon will play a central role. In the absence of genotypic mutations, the mean fitness is 1 −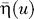 , where 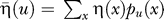 , and *p_u_*(*x*) denotes the probability that a cell with a phenotypic mutation rate *u* uses *x* amino acids to synthesize abnormal proteins. Genotypic mutations reduce this mean fitness. Because we ignore back mutations, at mutation–selection balance the mean fitness is reduced by the factor (1 − *μ*)*^n^*, and this factor is independent of the actual fitness of the mutants. This is a special case of Haldane's principle for the mutation load (see the section Mutation Rates at Equilibrium. The mean fitness at mutation–selection balance is given by

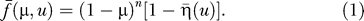



As a consequence, we get the following formula for the cost *C_u_* of phenotypic mutations, which is defined as the difference between the expected mean fitness of a population with and without phenotypic mutations:

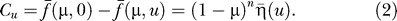



Similarly, the cost *C_μ_* of genotypic mutations is

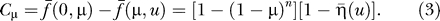



### Evolving Mutation Rates

We allow the mutation rates *μ* and *u* to evolve. Our aim is to derive approximations for the mutation rates that evolve in the long run. For this purpose, it is convenient to consider *μ* and *u* as quantitative traits with values between 0 and 1. Both traits have the potential to evolve due to new mutations which are assumed to occur at constant rates *π_μ_* and *π_u_*, respectively. These mutations will primarily increase the genotypic and phenotypic mutation rates, but some will decrease them. Because the precise form of these mutation distributions does not enter our approximate formulas given here, we introduce them only in the context of our computer simulation model below. Mutation is counteracted by selection because, on average, cells with a higher mutation rate have a lower fitness. Applying Haldane's principle, it can be shown (see the section Mutation Rates at Equilibrium) that the evolved genotypic mutation rate,μ̂, can be approximated as

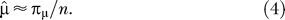



Similarly, the phenotypic mutation rate equilibrates to a value,*û*, which is obtained (approximately) by solving the equation

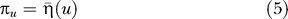
for *u*. If, as is likely the case, 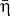 is monotone increasing, this solution*û* is uniquely determined. By taking the ratio of Equation 4 and Equation 5, and performing a simple rearrangement, we obtain

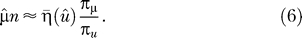



The term 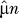 gives the number of mutations per genome per replication and is surprisingly constant for a wide range of organisms [1]. The rates *π_μ_* and *π_u_* at which the genotypic and phenotypic mutation rates are changed will primarily depend on the number of genes (and their length) involved in DNA replication and protein synthesis, respectively. In our model, for a given set of parameters, genotypic and phenotypic mutation rates evolve independently. Although we focus on the evolution of phenotypic mutation rates, for the analysis of our simulations it proved useful to also keep track of the genotypic mutation rate. Its equilibrium value is independent of the cost function *η* and can be used to estimate the effectiveness of selection (i.e., the drift–selection equilibrium value of*f̄*) for given population size and given values of *π_u_* and *π_μ_* .

A more detailed expression for the evolved phenotypic mutation rate can be obtained by approximating the cost function *η*(*x*), presumably concave, by a linear one. Thus, let us assume henceforth *η*(*x*) = *cx*, so that fitness decreases linearly with the number of amino acids used to synthesize erroneous proteins. Here, *c* measures the costs per codon to synthesize an abnormal protein. As a consequence, we can write 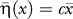 . In the Discussion we will address how nonlinear cost functions might affect our results.

Since, according to our model, a cell produces proteins until exactly *y_i_* functional proteins of gene *i* are synthesized, the number *x_i_* of nonfunctional proteins produced during the process follows the negative binomial distribution *NB*(*y_i_*,1 − *u_i_*). Here, 1 − *u_i_* gives the probability of a successful protein synthesis. The expected value of *x_i_* is 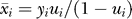 . Hence,*x̄*, the expected value of *x*, is given by

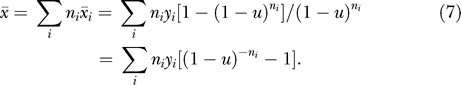
For *un_i_* < 1, we obtain

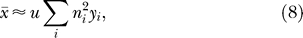
and 
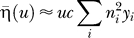
 . Therefore, Equation 6 can be rearranged to

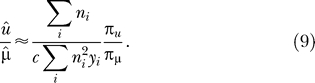



This illustrates nicely the factors that determine the ratio of the evolved genotypic and phenotypic mutation rates. This ratio depends on (i) the effective genome size, ∑*_i_n_i_*, (ii) the average total cost of abnormal protein synthesis, 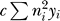 , and (ii) the ratio at which mutations of the two mutation rates occur. Below, we will use computer simulations to complement these analytical considerations.

### Simulating the Evolution of Mutation Rates

We simulate the evolution of phenotypic and genotypic mutation rates based on the model introduced above. The main difference is that we use a finite (effective) population of size *N* = 10^4^ and that we specify our mutation distributions for the mutation rates. Each generation, *N* organisms are selected (with replacement) for reproduction with probabilities proportional to their fitness (Wright-Fisher model of drift and selection). Fitness is calculated as 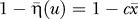 , where*x̄* gives the expected amount (in amino acids) of abnormal proteins. Protein length and abundances are taken from S. cerevisiae (see Materials and Methods). The number of expected abnormal proteins is calculated according to Equation 7. To avoid the fixation of genotypic mutants for high genotypic mutations rates at the beginning of the simulations, we set the fitness of genotypic mutants to zero. As predicted by the theory, the fitness of the genotypic mutants does not affect the equilibrium mutation rates (see the section Effect of Initial Values and Parameters on the Simulation Results).

The initial population is homogeneous with equal phenotypic and genotypic mutation rate. To allow the evolution of mutation rates, we change (mutate) *u* and *μ* with probabilities *π_u_* and *π_μ_*, respectively. Unless otherwise mentioned, we assume *π_μ_* = *π_u_* = 10^−4^. In the section Effect of Initial Values and Parameters on the Simulation Results, we show that changes in the initial values of *u* and *μ* do not affect the results of our simulations and changes in *π_u_* and *π_μ_* affect them as predicted by the theory. Since beneficial mutations, that is, mutations decreasing the mutation rate, are generally rare, we increase the mutation rate with a probability of 0.99 (conditional on a mutation event). In this case, we draw a new phenotypic mutation rate from a beta distribution *B*(*a* = 1,*b* = 9) on [*u*,2*u*] (or on [*μ*,2*μ*] for genotypic mutation rates). Hence, the average increase is 10% and small changes are more frequent than large ones. Similarly, in case of a decrease, we draw the new mutation rate from a reflected beta distribution *B*(*a* = 1,*b* = 9) on [0, *u*] (or [0, *μ*]). Hence, small changes are again more likely than large ones.

During a simulation run, we kept track of the ancestry of each individual. After 4 × 10^6^ generations, we calculate the most recent common ancestor of the population and determine its line of descent. We can use this line of descent to observe the evolution of phenotypic and genotypic mutation rates. For each parameter combination, we conducted ten runs that differ only with respect to the seed for the random number generator. Based on the ten trajectories, we compute an expected evolutionary trajectory for each parameter combination by calculating the geometric average of *μ* and *u*.

Figure 1 shows these average trajectories of *u* and *μ* for seven sets of simulations that use different values for *c*, ranging from 1 × 10^−16^ to 1 × 10^−10^, with increments of one order of magnitude. For all seven cost values, the genotypic mutation rates (lower lines) show essentially identical behavior. They decrease from the initial value of 1 × 10^−7^ to about 1.01 × 10^−9^ which leaves the cost of genotypic mutations at about *C_μ_* = 2.84 × 10^−3^.

**Figure 1:**
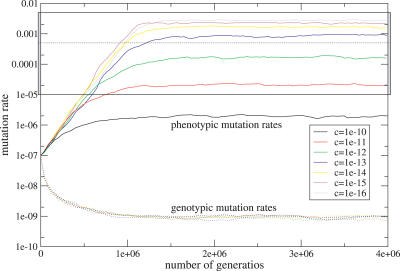
Evolving Phenotypic and Genotypic Mutation Rates The evolution of phenotypic (solid lines) and genotypic (dotted lines) mutation rates is shown. For each of seven different values for the cost of erroneous proteins, *c*, we conducted ten simulation runs and calculated an average evolutionary trajectory (see text for more details). As expected, only the phenotypic mutation rate is affected by changes in *c*. Near *u* = 2 × 10^−3^ we observe an upper limit for the phenotypic mutation rate. Even large changes in *c* affect the phenotypic mutation rate only marginally (compare grey and brown lines). This (effective) upper bound for *u* is the result of a rapid, nonlinear increase in abnormal proteins as a function of *u* (see Figure 2). The (grey) box indicates the possible range of phenotypic mutation rates (1 × 10^−5^ – 5 × 10^−3^, according to Parker [5]) with 5 × 10^−4^ (dashed line) as a commonly used estimate for the global error rate.

**Figure 2:**
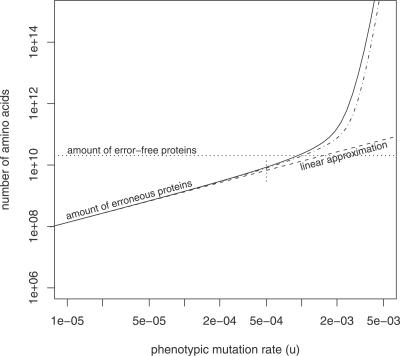
The Amount of Erroneous Proteins as a Function of the Phenotypic Mutation Rate, *u* The solid curve shows the expected number*x̄* of amino acids required to synthesize abnormal proteins according to Equation 7 with values for *n_i_* and *y_i_* from yeast (see Methods and Materials). The dash-dotted curve shows the total number 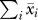 of abnormal proteins. For better comparison, we scaled the number of proteins so that the dash-dotted and solid curves meet at *u* = 10^−5^. The dashed line shows the linear approximation to*x̄* (see Equation 8). The dotted line indicates the amount (in amino acids) of functional proteins in a yeast cell, which equals 2.029 × 10^10^. Near *u* = 5 × 10^−4^ (the estimate for the global phenotypic mutation rate), the linear approximation begins to deviate noticeably from the exact value. A doubling of *u* at this point would result in more erroneous than error-free proteins. Another doubling would result in more than seven times as many erroneous than error-free proteins. This nonlinear increase is also observed if one considers the number of abnormal proteins (dash-dotted curve).

The equilibrium value of the phenotypic mutation rate depends strongly on the cost of abnormal proteins. For the given values of *n* = 2.81 × 10^6^ and 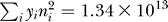 (see Materials and Methods) and because we assume *π_μ_* = *π_u_*, Equation 9 predicts 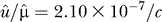 . For *c* = 10^−10^, this equals 2.10 × 10^3^ and is indeed very close to the observed value of 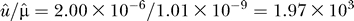 (see black curves in Figure 1). For this set of simulations, the phenotypic mutation rate evolves to a level at which the cost of phenotypic mutations is *C_u_* = 2.67 × 10^−3^, which is very close to *C_μ_*.

As expected, a decrease in the cost of abnormal proteins, *c*, results in an increase of the phenotypic mutation rate. But, apparently, there is an upper limit for phenotypic mutation rates above which a further decrease of *c* does not increaseμ̂ much more (compare brown and grey curves in Figure 1). Also, for very small *c*, Equation 9 becomes inaccurate. For example, if *c* = 10^−13^, we get 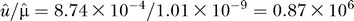 instead of 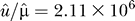 .

We can explain both observations by considering the average number*x̄* of amino acids required to synthesize nonfunctional proteins (Equation 7) and the total number 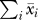 of nonfunctional proteins. For brevity and in distinction to the number of nonfunctional proteins, 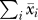 , we henceforth refer to 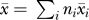 as the *amount* of abnormal proteins. (We use the number of amino acids as the unit for the amount of proteins.) Figure 2 displays these quantities as functions of *u* (solid line for*x̄*, dash-dotted line for 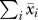 ) as well as the linear approximation (Equation 8) to*x̄* (dashed line). As one can see, the linear approximation becomes inaccurate if *u* > 5 × 10^−4^, and*x̄* begins to grow exponentially with *u*. Consequently, even a small increase in *u* will cause a tremendous increase in*x̄*. Even if abnormal proteins are not very costly, the rapid increase in*x̄* prevents a further increase of *u*.

We used 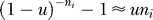 to linearize*x̄*. This approximation is only accurate if *un_i_* is sufficiently small. For a protein length of about 890 amino acids (see below for an explanation why we chose 890) and a phenotypic mutation rate of 5 × 10^−4^, we have *un_i_* = 0.445, which is apparently too large for the approximation to be accurate. For *n_i_* = 890 and *u* = 5 × 10^−4^, the exact value for 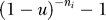 is 0.56; this illustrates the difference between the true value of*x̄* and its linear approximation at *u* = 5 × 10^−4^. It is important to emphasize that the observed upper bound for *u* is a consequence of the protein length distribution and the expression profile of the organism, as we will see below.

### Components of*x̄*

It would be interesting to know the length and expression level of the genes that contribute most to the amount of abnormal proteins in a cell. For a given phenotypic mutation rate, it is easy to calculate 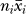 , the amount (in amino acids) of erroneous proteins that were produced from gene *i*. To determine which gene lengths and expression levels are most important for*x̄*, we calculate weighted averages of *n_i_* and *y_i_*. As weights, we use the amount of erroneous proteins that stem from gene *i*, that is, 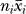 . Hence, we have

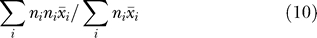
and

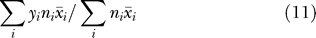
as indicators of the average protein length and expression level, respectively, that are most important for the amount of abnormal proteins in the cell. Since the expected number of abnormal proteins, 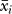 , depends on the phenotypic mutation rate, the weighted averages are functions of *u* as well. How they change as a function of *u* is shown in Figure 3. Interestingly, the weighted average of *n_i_* for very small *u* is about 890 and nearly twice as large as 496, the average protein length in yeast. Even more interestingly, this value increases suddenly as *u* increases beyond 5 × 10^−4^. For high values of *u*, phenotypic mutations are so frequent that it becomes essentially impossible to synthesize large proteins accurately. These large proteins dominate the cost of phenotypic errors. This can also be seen in the change of the weighted average of the expression level. For low mutation rates, the amount of erroneous proteins is dominated by highly expressed genes with a weighted average expression level of 2.67 × 10^5^ proteins per cell. This value begins to decrease at *u* = 5 × 10^−4^ because large proteins, instead of highly expressed proteins, begin to increasingly contribute to the amount of abnormal proteins in the cell.

**Figure 3:**
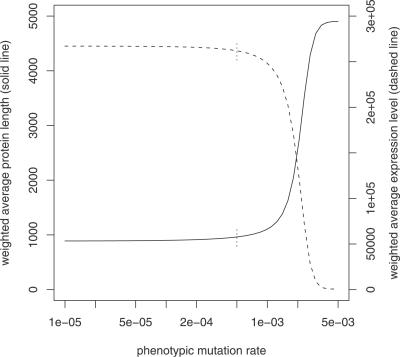
Weighted Averages of Protein Length (10) and Expression Level (11) To determine which protein and expression levels are most relevant for the amount of abnormal proteins in a cell, we calculated weighted averages of protein length (solid line) and expression level (dashed line). As weights, we used the amount of expected abnormal proteins, 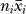 . For small phenotypic mutation rates, highly expressed proteins are most relevant for the amount of abnormal proteins in a cell. This begins to change at *u* = 5 × 10^−4^, when lowly expressed, large proteins begin to dominate*x̄*. Inaccurate protein synthesis makes it practically impossible to synthesize these larger proteins error-free.

For *u* = 5 × 10^−4^, the bulk of the amount of erroneous proteins comes from proteins that are noticeably larger than the average protein and are highly expressed. How much these proteins contribute to*x̄* compared with the rest of the genome can be seen in Figure 4. The solid line shows 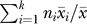 for 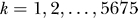 , that is, the cumulative contribution of each gene to*x̄*. Genes are sorted decreasingly by their contribution to*x̄*. It is obvious that only few genes contribute to most of the abnormal proteins in the cell. In fact, 5% (10%) of the genes contribute to 78.6% (87.5%) of the abnormal proteins in a yeast cell. The average length of these proteins is 927 (818) which confirms the conclusion from above that genes that contribute most to the amount of abnormal proteins are much larger than the average gene.

**Figure 4:**
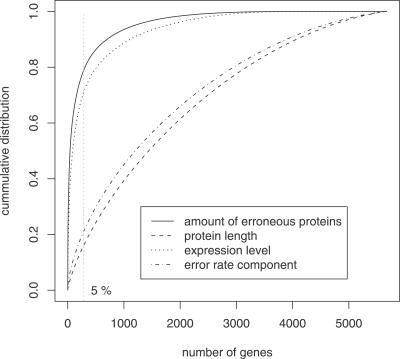
Relative Contribution of Each Gene to the Amount of Erroneous Proteins in Yeast if *u* = 5 × 10^−4^ The solid line shows the cumulative contribution of each gene to*x̄*. A steep increase, as seen here, indicates that few genes are responsible for most of the abnormal proteins in a cell. We also plot the cumulative distribution for the three components of 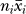 : protein length, *n_i_* (dashed line), number of functional proteins, *y_i_* (dotted line), and expected amount of erroneous proteins to synthesize one error-free protein, 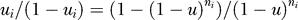 (dash-dotted line). The curvature of the solid line is similar to the curvature of the dotted line. Hence, most of the abnormal proteins stem from few genes because these genes are also expressed at a very high level.

From Equation 7 we know that *n_i_*x̄*_i_ = n_i_y_i_*(1−(1−*u*)^*n_i_*^)/(1−*u*)^*n_i_*^ and can distinguish three components: (i) the protein length, *n_i_*, (ii) the expression level, *y_i_*, and (iii) the expected number of erroneous proteins that have to be synthesized to get one error-free protein, 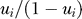 , with 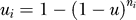 . Which of these components is primarily responsible for the fact that only few genes contribute to most of the abnormal proteins in a cell? To answer this question, we can compare the dashed, dotted, and dash-dotted lines in Figure 4 which show how unevenly genes contribute to each of the three components (for *u* = 5 × 10^−4^). For example, the dotted line shows 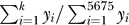 for 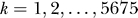 . From the three components, only the expression level (dotted line) shows a curvature similar to the solid line. Hence, the fact that few genes contribute to most of the abnormal proteins in a cell is due to differences in expression levels rather than differences in protein length.

### Adaptation of Highly Expressed Proteins

If most of the abnormal proteins in a cell are synthesized by few, highly expressed proteins, the cell could reduce the cost of phenotypic mutation rates considerably by decreasing the phenotpyic mutation rate for these few genes. In fact, highly expressed genes are special in many ways. They use preferred codons more frequently than “normal” genes [28] and evolve more slowly [29]. The usage of preferred codons conveys several advantages, among them is a more efficient [30–32] and accurate [33–35] translation. As argued by Drummond et al. [29], the slow rate of evolution of highly expressed genes might be the result of selection for translational robustness, that is, the ability of proteins to work properly despite amino acid substitutions [15,29]. The effect of preferred codon usage and translational robustness are conceptually very different. The usage of preferred codons reduces the phenotypic mutation rate by reducing the amino acid substitution rate, while an increase in translational robustness reduces the phenotypic mutation rate by improving a protein's ability to withstand the effect of amino acid substitutions. Let us first consider translational robustness.

### Selection for translational robustness

Let *u*_aa_ denote the amino acid substitution rate. Together with the robustness of a protein against amino acid substitutions it determines the protein's phenotypic mutation rate *u_i_*. According to Bloom et al. [36], the probability that a protein retains its wild-type structure after *m* amino acid substitutions is given by

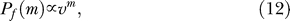
where *v* denotes the average neutrality to amino acid substitutions (m-neutrality), that is, the average probability that a protein will be unaffected by (“neutral” to) an additional amino acid substitution. In the following we make the conservative assumption that a protein is functional if it is able to fold into its wild-type structure and that the wild-type sequence always folds into its wild-type structure, i.e., that 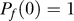 . Consequently, 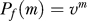 is the probability that a protein exposed to *m* amino acid substitutions is functional.

A protein contains *m* amino acid substitutions with probability

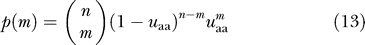
and is therefore functional with probability

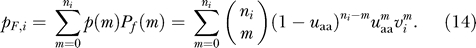



The probability to synthesize a nonfunctional protein from gene *i* is given by


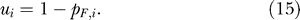


For protein *i*, the average number of nonfunctional proteins is given by 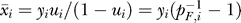 , and the overall average amount of nonfunctional proteins in a cell is given by

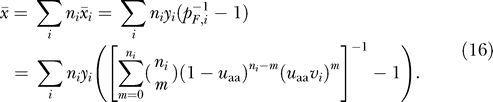



In comparison with Equation 7, we note that 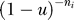 , the term that is responsible for the rapid, nonlinear increase of*x̄*, has been replaced by a sum over the number of amino acid substitutions. Since *n_i_* is usually much larger than the number of amino acid substitutions, we have *n_i_* ≈ *n_i_* − m for relevant *m* values and can approximate the sum very accurately by

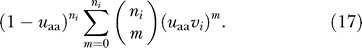



We see that the term that caused the dramatic increase in the previous model also appears in this model, which considers translational robustness. The phenotypic mutation rate *u* is replaced by *u*_aa_ and the term 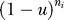 multiplied by a factor that depends on the protein's m-neutrality *v_i_*. Analogous to our previous observations, we can expect a nonlinear increase of*x̄* for *u_aa_* > 5 × 10^−4^.

In theory, but not in practice, it is possible to reduce the phenotypic mutation rate to zero by increasing *v_i_* to 1 for all proteins. In practice, an upper limit for *v_i_* is given by the function and stability of the protein. In our simulations, this upper limit is set by a prior distribution for the values of *v_i_*. Very high values for *v_i_* will be possible but unlikely. Given this (soft) upper limit for m-neutralities, we can, for a given amino acid substitution rate, ask which proteins will be selected for translational robustness (large m-neutralities) and what amount of abnormal proteins can be expected.

We present simulations in which m-neutralities are drawn from a beta distribution, *B*(*v*|*a*,*b*) ∝ *v^a^* ^−1^ (1 − *v*)*^b^* ^−1^ with *a* = 16.95 and *b* = 20.72, which has variance 0.0064 and mean 0.45, reflecting the mean and variance of m-neutralities of seven proteins estimated by Bloom et al. [36]. We want to emphasis that the quantitative results, in particular the relative changes as a function of *u*_aa_, are not affected when other (reasonable) prior distributions are used. We obtained similar results for a beta distribution with mean 0.5 and variance 0.02 and for corresponding normal distributions (truncated to the interval [0, 1]). Even though the absolute value of*x̄* is smaller for prior distributions that allow larger m-neutralities, the relative changes remain the same.

Mutations generate m-neutralities *v_i_* for each protein from the prior distribution. Selection determines if an m-neutrality reaches fixation and subsequently the eventual distribution of *v_i_* after many generations of selection. By how much selection has caused a protein's m-neutrality to deviate from the prior distribution can be expressed in terms of the log likelihood (LL) of the *v_i_* values under the prior distribution, log(*B*(*v_i_*|*a*,*b*)) ∝ (*a* − 1) log (*v_i_*) + (*b* − 1) log (1 − *v_i_*). If selection leads to a significant increase of a protein's m-neutrality, then the LL of this m-neutrality will be very low. The average LL for a cell's proteins is given by

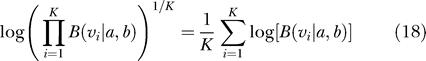
and can be used to quantify the extent of selection for translational robustness that the proteins of a cell were exposed to. As we will see, the LL of the m-neutralities (after selection) decreases substantially for *u*_aa_ > 5 × 10^−4^ and indicates the intensified selection for translational robustness.

We use a drift-selection model based on the fixation probability in a Moran process to determine the *v_i_* values at mutation–selection balance (the post-selection *v_i_* distribution). For a given amino acid substitution rate, we initialize *v_i_* for all proteins by setting it equal to the mean of the prior distribution and calculate*x̄*. For every protein (gene) *i*, we draw a new *v_i_* from the prior distribution and calculate the new*x̄* that reflects this change, 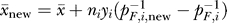 . For computational reasons and since the binomial distribution has most of its weight at small values of *m*, we truncate the summation over *m* in Equation 14 to the smallest integer larger than 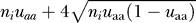 (i.e., *m*-values that are more than four standard deviations away from the mean are ignored; the probability of getting *m*'s above this threshold is less than 2 × 10^−3^). We accept the new *v_i_* with probability (1–1/*r*)/(1–1/*r^N^*), which corresponds to the fixation probability in a Moran process of a mutant with relative fitness *r* = *f*_new_/*f* in a population of size *N*. We use *N* = 10,000 and 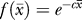 , with *c* = 10^−9^. Here, we cannot use the fitness function 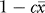 as before, because it might lead to negative fitness values. In the previous section, we chose 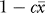 because of its analytical tractability and its similarity to the cost of genotypic mutations (see Equation 1). We did not have to worry about negative values for 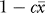 since *u* could evolve freely and selection caused *u* to converge to levels where 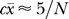 < 1. In this section, *u*_aa_ is constant and the adaptation of individual proteins cannot reduce*x̄* arbitrarily (there are upper limits to *v_i_*). Note that 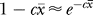 for small 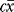 . Hence, our results from the previous section, where we used 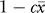 as cost function also hold for the cost function 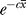 .

For each *u*_aa_, we report the average of 20 simulations, which differ only with respect to the seed for the random number generator. For each simulation, we sequentially conducted 500,000 updates of each protein as described above. We then analyzed which proteins were selected for translational robustness by calculating the LL. We also analyzed to which extent*x̄* is reduced by increasing *v_i_* and how an increase in *u*_aa_ affects the selection for translational robustness.

Figure 5 summarizes the results of our simulations. The top and the middle panels show*x̄* and the average LL (18) after selection as a function of *u*_aa_. Similar to the previous section, we notice a dramatic increase of*x̄* for *u*_aa_ > 5 × 10^−4^. This is not surprising, considering the mentioned analytical similarities between Equations 7 and 16. For amino acid substitution rates above 5 × 10^−4^, the cell has difficulties to prevent the increase of*x̄*. This is also evidenced by the decline of the LL. The lower panel in Figure 5 shows the change in the average LL of three sets of 100 proteins. The three sets of proteins are given by the 100 proteins with the largest *n_i_*, *y_i_*, and 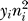 , respectively. As expected, proteins with large 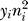 values are more effectively selected for large m-neutralities than large or highly expressed proteins and, accordingly, have the smallest LL. With increasing *u*_aa_, large proteins contribute more to the amount of abnormal proteins and the corresponding LL decreases more rapidly than for the other two groups of proteins. In Figure 6 we show the m-neutralities of individual proteins for three amino acid substitution rates. It illustrates the intensified selection for large m-neutralities in large proteins.

**Figure 5:**
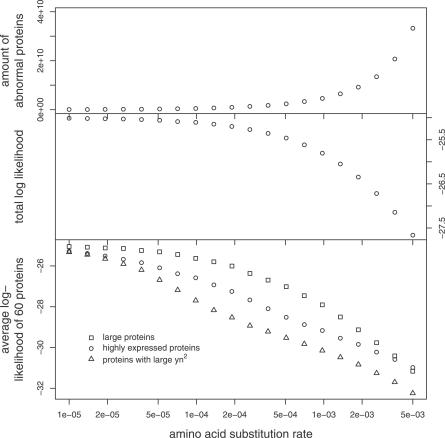
Selection for Translational Robustness as a Function of Amino Acid Substitution Rate The top panel shows the average amount of abnormal proteins,*x̄*, after 500,000 cycles of mutation and selection (see text for more detail). The mid-panel shows the average log-likelihood (LL) of the m-neutralities after selection. For *u* ≥ 1.92 × 10^−5^, the average LLs are significantly lower than expected by chance. The 5 (0.1) quantile is given by −25.024 (−25.040). The total LL decreases noticeably for *u*_aa_ > 5 × 10^−4^. The lower panel shows the average LL of three groups of 100 proteins. We consider the 100 largest proteins, the 100 most highly expressed proteins, and the 100 proteins with the largest 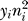 . For 100 proteins, the 5% (0.1%) quantile for the average LL is −25.117 (−25.215). The lower panel shows that the extent of selection for translational robustness increases nonlinearly for large proteins whereas it increases approximately linearly in the other two groups of proteins.

**Figure 6:**
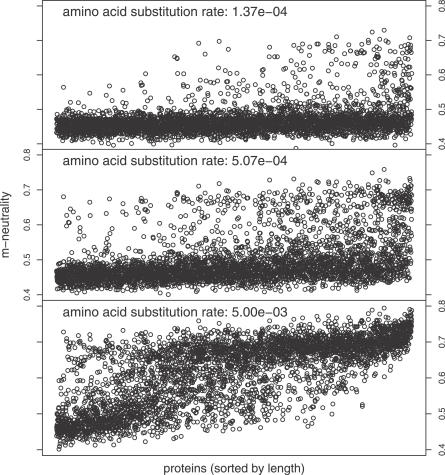
m-neutralities, *v_i_*, After Selection for Three Different Amino Acid Substitution Rates *u_aa_* The data points are sorted according to the length of the proteins. The increased effectiveness of selection for higher m-neutralities in large proteins for high amino acid substitution rates is clearly visible.

The simulations conducted in this section implicitly assume that the population is homogeneous and that one mutant appears at a time and either goes extinct or gives rise to another homogeneous population. Hence, every organism in these simulations represents a homogeneous population of size *N*. This organism is of course also the MRCA of this population. We can compare its fitness, 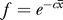 , with the fitness, 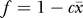 (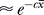 for 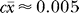 ), of the MRCA in our previous simulations to speculate on what would happen if we allowed *u*_aa_ to change here as well. Here, where *u*_aa_ was held constant, the fitness of the organism converged to fairly small values compared to the equilibrium values of *f* ≈ 0.995 in our previous simulations which allowed changes in *u*. For *u*_aa_ = 1 × 10^−5^, where selection for higher m-neutralities is insignificant, *f* = 0.938, and *f* is much lower for larger phenotypic mutation rates (e.g., *f* = 0.090 for *u_aa_* = 5 × 10^−4^). This suggests that if *u*_aa_ is able to evolve freely to equilibrium values of *f* ≈ 0.995, then selection for translational robustness will be insignificant. Simulations in which we mutated *u*_aa_ as described in the previous section confirmed this expectation. No significantly elevated m-neutralities evolved (unpublished data).

This was not the case in simulations with few (e.g., ten) genes, where the LL of highly expressed proteins converged to significantly lower values. Apparently, if there are many genes and if *u*_aa_ is in mutation–selection balance, the m-neutralities of individual proteins do not contribute enough to allow selection for higher m-neutralities. But as we have seen above, if *u*_aa_ is constant, selection for larger m-neutralities can reduce*x̄* to some extent. Hence, if *u*_aa_ is above its mutation–selection-balance value, then significantly higher m-neutralities will evolve and decrease the phenotypic mutation rate by decreasing the effect of amino acid substitutions.

### Selection for Preferred Codons

Besides increasing the translational robustness of certain proteins, a cell can also use preferred codons to decrease the phenotypic mutation rate *u_i_*. This would actually decrease the amino acid substitution rate and is therefore conceptually different from translational robustness, which reduces the effect of amino acid substitutions but not their occurrence. Considering codon usage, the amino acid substitution rate *u*_aa_ has two components, a ribosomal component *u_r_* and a codon-based component, *u_c_*. We assume that *u*_aa_ = *u_r_u_c_* for preferred codons and that *u*_aa_ = *u_r_* for nonpreferred codons. Preferred codons are more accurate than nonpreferred codons, hence, *u_c_* < 1. In this section we ignore translational robustness, i.e., *u* = *u*_aa_. A protein of length *n_i_* that uses 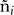 preferred codons synthesizes a functional protein with probability 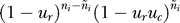 . The average amount of abnormal proteins is given by

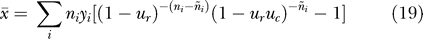


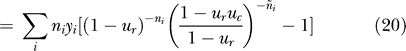



Again, we notice similarities between Equations 7 and 19 and can expect a rapid increase of*x̄* for *u_r_* increasing above 5 × 10^−4^ .

We conducted simulations analogous to those investigating the effect of translational robustness. We calculate*x̄* according to Equation 19 with *u_c_* = 0.1. Each time we mutate the number of preferred codons, we increase 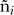 by one with probability 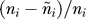 (the fraction of nonpreferred codons in the gene). We decrease 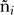 by one with probability 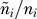 . After changing 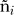 , we calculate 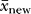 and accept the new 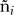 as described in the section Selection for Translational Robustness. We report the average of 20 simulations for each *u_r_*. Each simulation was terminated after 2 × 10^7^ sequential mutations (not necessarily fixation) of 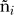 .

Figure 7 shows the results of our simulations. Analogous to Figure 6, we plot the equilibrium fraction of preferred codons, 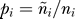 , for each gene for three ribosomal amino acid substitution rates *u_r_*. For *u* = 1.37 × 10^−4^, only few genes evolve a major codon bias of *p_i_* > 0.6. The gene with the largest codon bias of about 0.8 encodes for the protein with the largest 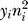 and contributes 5.7% to the total amount of functional proteins and 7.9% to 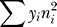 .

**Figure 7:**
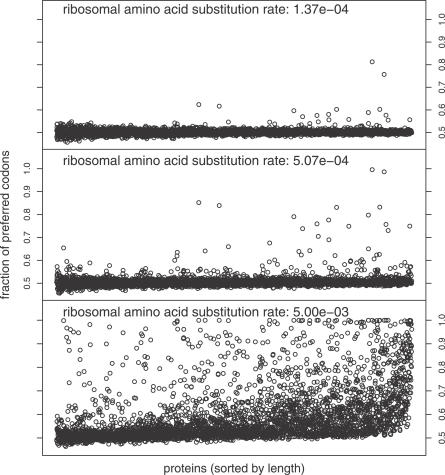
Fraction of Preferred Codons 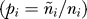 After Selection for Three Different Ribosomal Amino Acid Substitution Rates *u_r_* The genes are sorted according to the length of the protein. For *u_r_* = 1.37 × 10^−4^, selection introduces a codon bias only for few genes (compare with m-neutralities in Figure 6). An increase in the phenotypic mutation rate leads to more intensive selection for preferred codons in large genes than in small genes.

For *u_r_* > 5 × 10^−4^, large proteins begin to contribute more to the amount of abnormal proteins and selection increases the codon bias of large proteins. Similar to our observation in Figure 2, the codon bias cannot prevent the drastic increase of*x̄* for *u_r_* > 5 × 10^−4^. As before, no significant codon bias evolved if we allowed *u_r_* to change as well.

Comparing Figure 6 with Figure 7, we notice that selection for translational robustness results in a more distinct bias in *v_i_* than what we observe for *p_i_* after selection for preferred codons. In the next section we compare the two mechanisms to identify the source of this difference.

### Similarities between Preferred Codons and Translational Robustness

In our simulations, the two mechanisms differ in the way *p_F,i_*, the probability of synthesizing a functional protein, is calculated and in the way it is mutated.

For the translational robustness model, we calculate *p_F,i_* as

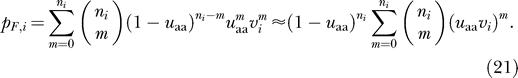



The approximations are accurate as long as proteins with many amino acid substitutions are rare. For the preferred codon model, we have

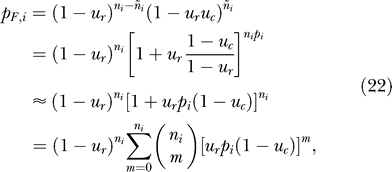
where we used 1 + *u*_*r*_/(1 − *u*_*r*_)≈1 + *u*_*r*_ and [1 + *u_r_*(1 − *u_c_*)]*^p_i_^*≈1 + *u_r_p_i_*(1 − *u_c_*), which are reasonable approximations if *u_r_* is small. The analogy between Equation 21 and Equation 22 is obvious. In theory, the m-neutralities, *v_i_*, can range from 0 to 1. In our simulations, for the chosen prior distribution, *v_i_*'s larger than 0.8 are rare. The m-neutralities *v_i_* are analogous to the term *p_i_*(1 – *u_c_*) in the preferred codon model. Since *p_i_* can range from 0 to 1, the two mechanisms can reduce the amount of abnormal proteins equally well if *v*_max_ = 1 – *u_c_*, where *v*_max_ denotes the upper limit for m-neutralities. In our simulations, we have *v*_max_ ≈ 0.8 < 1 – *u_c_* = 0.9. Hence, we would expect lower*x̄* values in the preferred codon model. This is not the case. For example, for *u*_aa_ = 10^−5^,*x̄* converged to 6.4 × 10^7^ in the translational robustness model, whereas*x̄* converged to 7.3 × 10^7^ in the preferred codon model. Hence, we have to consider the way in which the *p_F_*_,*i*_'s are mutated to understand this result.

In the translational robustness model, *v_i_* is sampled from a prior distribution. The new *v_i_* value is independent of the previous one. Hence, large changes of *v_i_* and, consequently, of *p_F,i_* are possible. In the preferred codon model, the number of preferred codons can only change in increments of one, and corresponding changes of *p_F_*_,*i*_ and*x̄* are small. The small changes in *p_i_* and, therefore, in *p_F,i_* allow the evolution of noticeable codon biases only in genes that produce large amounts of abnormal proteins. In the translational robustness model, large changes in the *p_F,i_*'s are possible, and they have a higher fixation probability.

Take, for example, the protein with the largest value of 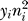 . It is 918 amino acids long, and a change of 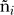 from 459 to 460 increases *p_i_* from 0.5 to 0.501 (by 0.2%). This small change reaches fixation only if the costs of abnormal proteins from this gene are very large. Changes larger than this are frequent in the translational robustness model and have a higher probability of fixation.

## Discussion

A functional protein machinery, built from genetic information, is central to every living organism. Surprisingly, the decoding of genes into amino acid sequences is fairly inaccurate. Errors (phenotypic mutations) occur several orders of magnitude more frequently than during DNA replication. The frequency of errors depends on the codon and its context (see Table 1).

In this paper, we have explored the evolution of phenotypic mutation rates. In our model, a cell maintains protein synthesis until a certain number of functional proteins are present. Depending on the phenotypic mutation rate, *u*, a certain number of amino acids, *x*, are “wasted” in erroneous proteins and reduce the fitness of the organism by *η*(*x*). For simplicity, we used a linear cost function *η*(*x*) = *cx*. With genomic and proteomic data from S. cerevisiae [24,25], we discover (a) an effective upper bound for the phenotypic mutation rate, (b) that most of the abnormal proteins stem from genes that are highly expressed and substantially larger than the average yeast protein, (c) that an average phenotypic mutation rate of *u* = 5 × 10^−4^ is at a value where *x* begins to increase dramatically as a function of *u* and large, lowly expressed genes begin to contribute substantially to the amount of abnormal proteins, and (d) that an increased codon bias or translational robustness in highly expressed genes can reduce the amount of abnormal proteins but cannot stop the dramatic increase for amino acid substitution rates above 5 × 10^−4^.

To what extent do our results depend on the assumption that *η*(*x*) is linear and that gene expression is maintained until a certain number of functional proteins are present? Dekel and Alon [37] found a convex increase of the cost of protein synthesis with the amount of proteins synthesized. Considering this and that aggregates of misfolded proteins are, in a concentration-dependent way, toxic to cells [27,38], we can expect the cost of erroneous proteins to increase faster than linear with the amount of erroneous proteins produced. A nonlinear *η*(*x*), however, would only affect the position of the upper bound for *u*, which we observed in our simulations (see Figure 1). For a nonlinear cost function, we would expect this upper bound to be lower than what we have observed here, because of the nonlinear increase of *x* on top of the nonlinear increase of the costs of *x*. Results (b)–(d) are not affected by the shape of the cost function.

Let us now consider our assumption about the regulation of gene expression. The largest protein in the yeast genome is Mdn1p, a dynein-related AAA-type ATPase [24,39]. It is 4,910 amino acids long. For *u* = 5 × 10^−4^, only 12.8% of the synthesized proteins are error-free. To get the required number of 0.538 × 10^3^ error-free proteins [25], the cell has to synthesize 6 × 10^3^ proteins. This is not a tremendous burden considering that about 46,600 × 10^3^ functional proteins are synthesized in total. However, this number increases rapidly if *u* increases. Doubling or quadrupling *u* would require the synthesis of 72.7 × 10^3^ or 10,000 × 10^3^ proteins, respectively. It is unrealistic to assume that a cell will synthesize 10^7^ proteins to get 538 functional ones. But we can consider this rapid increase as an indication for the inability of the cell to synthesize this protein and would have to rephrase result (c) to account for our assumption about gene expression: (c′) a phenotypic mutation rate of *u* = 5 × 10^−4^ is at a value where it is still feasible to synthesize large proteins. Higher phenotypic mutation rates would make it impossible to synthesize large proteins.

Interestingly, if one considers the ability of the cell to synthesize a certain number of functional proteins after a certain number of synthesis attempts, an upper bound for *u* is also encountered. In this situation, however, this upper bound is not due to the increase in abnormal proteins and the associated cost but due to the inability of the cell to synthesize enough functional proteins. In such a situation the cost of abnormal proteins is largely irrelevant and the upper bound for *u* primarily a result of the protein-length distribution and not of the cost of abnormal proteins. Furthermore, in such a situation there is little selection pressure to reduce the phenotypic mutation rate much below this upper bound [16].

If the synthesis of large proteins is such a problem, why does the cell not synthesize many smaller proteins and assemble them after successful production? An intermediate check for proper folding (which equals proper function for most amino acid substitutions) would prevent the incorporation of nonfunctional subunits and reduce the probability of assembling a nonfunctional complex. In yeast, proteins with a length of about 1,000 amino acids are quite common. This suggests that the complexation of proteins much smaller than 1,000 amino acids constitutes a considerable challenge. For so many large proteins, it might be impossible to get the same biological function from a complex of smaller proteins. According to our model, an upper bound of 1,000 for the yeast protein length does not reduce the drastic increase by much. If we calculate*x̄* after removing all proteins from the dataset that are larger than 1,000 amino acids, we can still observe a rapid increase in*x̄* at *u* = 5 × 10^−4^; doubling (quadrupling) *u* would lead to a 2.4 (7.3)-fold increase in the amount of abnormal proteins. Therefore, partitioning extremely large proteins into protein complexes is not sufficient to avoid the negative effects of an increasing phenotypic mutation rate.

Instead of complexing large proteins, evolution could reduce the phenotypic mutation rate of individual proteins. The phenotypic mutation rate of individual proteins could be reduced by using preferred codons [33–35] or by increasing the translational robustness of proteins [15,29,40]. Our analysis shows that these two mechanisms have nearly the same potential to minimize*x̄* if *u_c_* is sufficiently small (i.e., if preferred codons are sufficiently more accurate than nonpreferred codons). One big difference between preferred codons and translational robustness is the way in which the trait is mutated. For preferred codon usage, it seems reasonable to assume that the number of preferred codons changes in increments of one, which leads to very small changes in the amount of abnormal proteins.

Considering translational robustness, little is known about how mutations change the translational robustness of a protein. In our simulations, we mutate the translational robustness of a protein by sampling it from a prior distribution, which allows for large changes. Alternatively, one can use models that allow only small changes in a protein's translational robustness. More empirical data on the translational robustness spectrum of proteins is necessary to develop a satisfying model.

The effect of incremental changes of the number of preferred codons on the amount of abnormal proteins is fairly small. An increase in the number of preferred codons by one increases the probability of synthesizing a functional protein only by a factor of (1 – *u_r_u_c_*)/(1 – *u_r_*). For *u* = 5 × 10^−4^ and *u_c_* = 0.1, this equals 1.00045. Since only few genes contribute much to the amount and number of abnormal proteins, this will lead to very small changes of*x̄* for most proteins.

As mentioned previously, preferred codons are also able to increase the rate of translation. Selection for faster translation (or higher expression level) could be responsible for the observed codon biases. Since the time it takes to synthesize *y_i_* functional proteins is proportional to *y_i_n_i_* and the amount of erroneous proteins is approximately proportional to 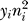 , it is possible to distinguish between the two sources of codon bias by comparing the observed codon bias in yeast with the predicted codon bias if selective forces were proportional to *y_i_n_i_* or 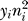 .

Further, a refined version of our preferred codon model that considers the genetic code and the actual amino acid sequence of each yeast protein could be used to estimate the cost of abnormal proteins and the amino acid substitution rate. For a given amino acid substitution rate, *u_r_*, an increase of the cost of abnormal proteins, *c*, increases the extent of codon bias but does not affect its distribution with respect to the protein length (the points in the top panel of Figure 7 would all move upward by an amount that is independent of *n_i_* since 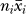 remains unchanged for constant *u_r_*). For given *c*, an increase of *u_r_* changes the extent of codon bias as well as the codon bias distribution with respect to the protein length (as seen in Figure 7, if *u_r_* increases, the codon bias of large proteins changes to a greater extent than the codon bias of small proteins since 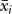 will increase more for genes with large *n_i_*). Hence, by choosing different values for *c* and *u_r_* and by comparing the resulting extent and distribution (with respect to *n_i_*) of codon biases with the extent and distribution of codon bias found in yeast, one can estimate the two parameters.

To experimentally measure the rate of amino acid substitutions during protein synthesis is notoriously difficult. Abnormal proteins are difficult to detect and usually degraded within minutes [27]. Experiments are usually limited to measuring the rate of specific substitutions at specific sites (see Table 1). One exception is work by Ellis and Gallant [4], who measured the rate of substitution of charged amino acids by uncharged amino acids. For many proteins such substitutions are detectable as satellite spots after 2-D gel electrophoresis. However, their method might fail to detect rapidly degraded abnormal proteins and is dependent on the number of codons at which charge substitutions can occur [4].

It would be highly desirable to be able to calculate the actual frequency of phenotypic mutations, that is, the frequency of deleterious amino acid substitutions during protein synthesis as opposed to the frequency of all (detrimental or not) amino acid substitutions. We can use our model together with data on the fraction of proteins that are abnormal and degraded rapidly [41,42] to calculate this. Schubert et al. [41] and Princiotta et al. [42] measured that in human cells about 33% and 25%, respectively, of newly synthesized proteins are rapidly degraded. The proteins are degraded mainly because of their inability to achieve a functional state [27]. Since these are values for human cells and might also include proteins that could not achieve a functional state despite error-free protein synthesis, we will use 15%–35% as the range for the fraction of proteins that are nonfunctional due to phenotypic mutations. In our model, *y* and*x̄* give the amount of functional and nonfunctional proteins synthesized, respectively. Hence the fraction of nonfunctional proteins synthesized due to phenotypic errors is given by 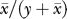 . According to our model (Equation 7) and the data from yeast (see Materials and Methods), 2.4 × 10^−4^ to 6.1 × 10^−4^ deleterious amino acid substitutions per codon would result in the synthesis of 15% to 35% nonfunctional proteins. Better estimates of the fraction of abnormal proteins in yeast would allow a narrowing of the calculated range.

## Mutation Rates at Equilibrium

Here, we derive our main analytical results on the magnitude of the genotypic and phenotypic mutation rates stated in the section The Model. We start by recalling Haldane's principle for an asexually reproducing population. This population is assumed to be sufficiently large so that random genetic drift can be ignored. The only evolutionary forces considered are selection and mutation. We assume that there is an optimal type (wild type) in this population. Its fitness is denoted by *W*_0_, the rate at which mutations to other types occurs is denoted by *U*, and back mutations are ignored. Then the mean fitness of the population at mutation–selection balance is given by 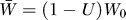 . This is obtained immediately from the recursion relation 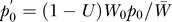 for the frequency *p*_0_ of the optimal type. The important, but simple point, first made by Haldane, is that the mean fitness is independent of the fitnesses of the deleterious types ([43], pp. 106–107).

This principle can be generalized to a large class of mutation patterns among possible types, and even to a continuum of possible types. It then states that in mutation–selection balance mean fitness 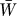 satisfies 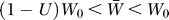 , where every type in the population is assumed to have the same mutation rate *U*. In addition, 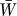 becomes asymptotically equal to 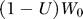 if the mutation rate *U* becomes sufficiently small. Detailed formulations as well as proofs can be found in ([43], pp. 127, 143–148). Again, the equilibrium mean fitness is, to first order in *U*, independent of the precise mutation pattern and of the fitnesses of the deleterious types.

Now we derive approximations forμ̂ and*û* in our model. We assume that the cost function *η* is linear, i.e., 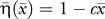 . Because of its complexity, we need a simplified model to make analytical progress. We identify all cells that have the same pair of mutation rates, (*μ*,*u*), and assign to them the average fitness 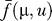 (see Equation 1) of a population of cells with these mutation rates. For given *μ* and applying Haldane's generalized principle to the trait “phenotypic mutation rate,” we get

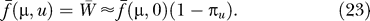



Rearrangement and use of Equation 8 yields the following approximation for the evolved phenotypic mutation rate at equilibrium:

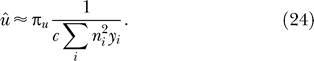



For the evolved genotypic mutation rate, we already have derived the approximation (Equation 4). The general theory [43], as well as numerical results (unpublished data), show that the above approximations forμ̂ and*û* are slight overestimates of the true values. Taking the ratio of Equation 24 and Equation 4, we obtain Equation 9.

## Effect of Initial Values and Parameters on the Simulation Results

To show the robustness of our results with respect to the initial conditions and the parameters, we conducted additional simulations analogous to the simulations presented in Figure 1. For Figure 1, we used *π_u_* = *π_μ_* = 10^−4^ and 10^−7^ as initial values of *u* and *μ*; genotypic mutations were lethal. The blue and violet lines in Figure 8 show that the initial values for *u* and *μ* and the fitness of the genotypic mutant do not change the equilibrium mutation rates at mutation–selection balance. The genotypic and phenotypic mutation rates will converge to the same equilibrium mutation rates as long as (a) the initial value for *u* is low enough so that 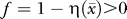 , and (b) the initial value for *μ* is low enough (or genotypic mutations deleterious enough) so that a fixation of genotypic mutants does not occur.

**Figure 8:**
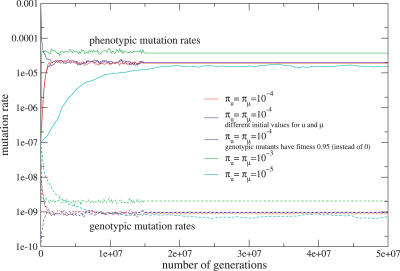
Evolution of Genotypic and Phenotypic Mutation Rates with Different Simulation Parameters (see the section Effect of Initial Values and Parameters on the Simulation Results) For all five parameter combinations, we use *c* = 10^−11^. Most simulations reach equilibrium within 1.5 × 10^7^ generations. For *π_u_* = *π_μ_* = 10^−5^, it was necessary to extend the simulations to 5 × 10^7^ generations. For simulations that end after 1.5 × 10^7^ generations, we plot straight lines thereafter. These lines serve as visual cues and are at the level of the last value.

We conducted simulations with different values for *π_u_* and *π_μ_*. The green and cyan lines in Figure 8 show the evolution of *u* and *μ* for *π_u_* = *π_μ_* = 10^−3^ and *π_u_* = *π_μ_* = 10^−5^, respectively. As expected, higher (lower) *π_u_* and *π_μ_* lead to faster (slower) evolution of *u* and *μ* and to increased (decreased) equilibrium values. The magnitude of this change is smaller than predicted by theory, e.g., Equation 9. This can be attributed to the finite population size, *N* = 10^4^. In finite populations, selection is inefficient for costs (C _u_,*C_μ_*) below a certain threshold. Note that from Equations 23 and 2 we have 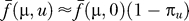 and 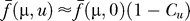 , respectively.

## Abbreviations

LL: log-likelihood
